# Six Months of Remote Patient Monitoring Is Associated with Blood Pressure Reduction in Hypertensive Patients: An Uncontrolled Observational Study

**DOI:** 10.1089/tmj.2022.0418

**Published:** 2023-08-04

**Authors:** William D. Frazier, Michael Beins, Joan DaVanzo, Steven Heath, Allen Dobson

**Affiliations:** ^1^ Verustat, Inc., Nashville, TN, USA.; ^2^ Dobson, Devanzo and Associates, LLC, Vienna, Virginia, USA.

**Keywords:** remote patient monitoring, hypertension, blood pressure reduction, monitoring frequency

## Abstract

**Background:**

Remote physiological monitoring (RPM) is a form of telehealth that measures vital signs at home and automatically reports the results to providers, thereby possibly improving chronic disease management. Medicare payment for RPM began in 2019. Two potential obstacles to RPM growth are the paucity of published clinical outcomes data and the Medicare requirement that monitoring be done at least 16 days per month to bill for the service. To help address these issues, we report the following uncontrolled observational study.

**Methods:**

A total of 1,102 consecutive patients enrolled in RPM were divided into four groups based on initial average mean arterial pressure (MAP) and into six groups based on the number of days per month MAP was measured. We report changes in MAP after 6 months of RPM as a function of initial MAP, and number of days per month MAP was monitored.

**Results:**

After 6 months of RPM, average MAP dropped from 97 to 93 (*p *< 0.01). This drop was greatest in the 50% of patients initially hypertensive. These patients saw average MAP reductions from 106 to 97 (*p *< 0.001) and became normotensive. Although MAP reduction was greatest the more frequently patients measured, significant reduction occurred in the hypertensive patients whether they measured more or less than 16 days per month (*p *< 0.001). No minimum threshold of measurements was found that predicted failure of RPM to lower MAP.

**Conclusions:**

RPM is associated with clinically and statistically significant reductions in average MAP in patients who were initially hypertensive. This benefit occurred irrespective of the number of days per month patients measured MAP.

## Introduction

The ability to monitor certain aspects of a patient's health from their own home has become an increasingly popular telehealth option. Remote patient monitoring (RPM) is a form of telehealth whereby technology is utilized to measure physiological parameters such as vital signs away from traditional clinical settings.^1^

Ideally, adding RPM to chronic disease management programs could improve an individual's quality of life and minimize health care costs by analyzing physiological parameters to allow earlier detection of decompensation and thereby reduce preventable emergency department visits and hospitalizations.^2,3^

In 2019, the Centers for Medicare and Medicaid Services (CMS) published three new current procedural technology (CPT) billing codes for RPM. These were 99453, 99454, and 99457 describing initial equipment set up, monthly data collection and interpretation, and monthly treatment management services, respectively. In 2020, a fourth code, 99458, was introduced allowing for additional 20-min monitoring episodes of RPM management services per month.^4^

One stipulation of these codes is that patients report measurements on at least 16 different days per month before RPM CPT codes 99453 and 99454 can be billed. No empirical data is available documenting that so many measurements are necessary to achieve clinical improvement. Such high thresholds may be a significant barrier discouraging provider and patient engagement with RPM.

Despite a proliferation of companies offering RPM services and an increasing number of patients being enrolled in these programs, there are few published reports detailing clinical outcomes, and, to our knowledge, none reporting changes in mean arterial pressure (MAP). We, therefore, report the results from an observational study of changes in MAP in the 6 months after the initiation of an RPM program as a function of initial MAP and of number of days MAP was measured per month.

### RESEARCH QUESTIONS

We sought to answer the following three questions: (1) Is there an association between RPM and changes in MAP, (2) does the initial MAP predict subsequent changes associated with RPM, and (3) is there an association between the number of days per month RPM measurements are reported and changes in MAP?

## Methods

The subjects of this observational study were 1,102 consecutive patients enrolled in the Verustat RPM program during the 1-year period from February 1, 2021, to January 31, 2022. The patients were followed until disenrollment from the program or until July 31, 2022.

Verustat is a for-profit RPM provider, and the initial enrollment date represents the company's first full month of operation, whereas the latter date was chosen so that every patient included in this report had the opportunity for at least 6 months of monitoring. The Verustat RPM model incorporates a care coordinator assigned to each patient. Monitoring equipment is delivered within 5 days of RPM enrollment and patients receive initial setup instructions by telephone.

In the 20% of patients who are unsuccessful using this approach, an in-person home visit is quickly arranged to complete equipment setup. Any day that patients fail to monitor, a care coordinator calls and sends text messages to remind them to use the monitoring equipment. If a measurement falls outside ranges specified by the ordering provider, the care coordinator again uses phone calls or texts to contact the patients and ask them to recheck the measurement. If it remains out of range, the health care provider is notified using a provider-specific communication protocol.

Changes in MAP in mm/Hg were the variable chosen for observation since it was monitored by the entire patient cohort. MAP measurements were obtained with A&D, Omron, or Indie Health blood pressure (BP) cuffs, and the results were automatically sent to a central monitoring portal by cellular signal or Bluetooth technology.

Month 1 was the 1st month in which a patient used RPM to measure MAP, and this initial value was considered the patient's baseline. Final MAP was defined as the last measurement reported in month 6 after RPM was begun. Measurements were reported between 0 and 31 different days per month. If a patient reported 0 measurements for 2 consecutive months, they were disenrolled from the program. To ascertain whether RPM was associated with changes in MAP, we compared the average initial MAP of the patient cohort with the average final value 6 months after RPM was begun.

We divided the initial average MAP measurements into quartiles (*n* = 275) so that quartile 1 (Q-1) represented the lowest average initial MAP, quartile 2 (Q-2) the next lowest, quartile 3 (Q-3) the next, and quartile 4 (Q-4) the highest average initial MAP. We analyzed the effect of initial BP quartile assignment on final MAP values.

Lastly, we divided the patient cohort into six groups based on the average number of days they transmitted MAP values to the portal each month. These groups were made up of 44 patients who averaged 0–7 measurements per month, 217 patients who averaged 8–15 measurements per month, 512 patients who averaged 16–24 measurements per month, and 329 patients who averaged >24 days of measurements per month.

We also divided the patients into a Medicare “compliant and billable” group that averaged 16 or more days per month of MAP measurements (*n* = 841) and a “noncompliant and nonbillable” group that averaged <16 days per month of measurements (*n* = 261). We analyzed how these different “number of times per month” measurement groups affected the changes in average MAP.

This observational study did not require informed consent since all the data were collected as a routine part of RPM services and since the data analyzed for this study contained no individual patient identifiers or protected health information. In addition, the results were reported as group averages and not as individual patient outcomes.

## Results

The 1,102 patients recorded an average of 28 measurements per month during the 6-month observation period. An average of 76% of the patients (*n* = 841) reported 16 or more days of measurements per month for each month of the study period, and, therefore, met the Medicare minimum threshold for billing RPM services. Slight fluctuations were seen in number of patients reporting measurements month over month because some patients occasionally missed measuring their MAP one month, but then resumed measuring the next month. For instance, month 6 included 1,098 patients who measured at least once, which is 99.6% of the 1,102 that measured at least once in month 1.

### MAP CHANGES FOR THE TOTAL PATIENT COHORT

**Fig. 1.**
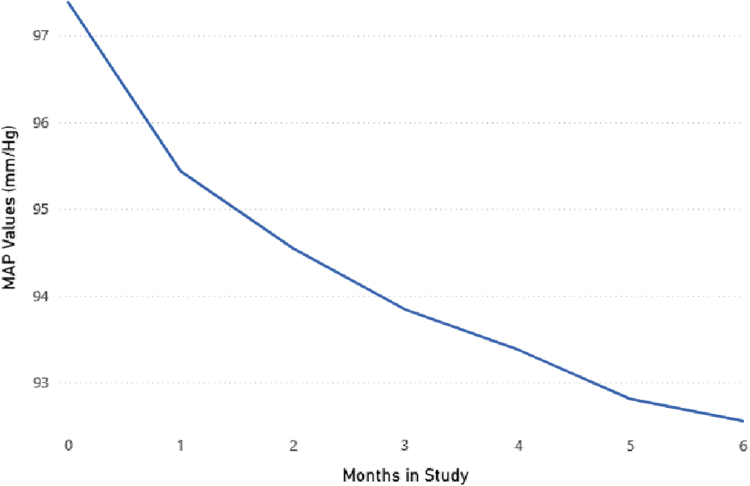
MAP changes (mm/Hg) for total patient cohort after 6 months of RPM. MAP, mean arterial pressure; RPM, remote physiological monitoring.

Average MAP decreased for the total patient cohort (*N* of 1,102) during the 6-month observation period. Initial average MAP was 97 mm/Hg and dropped to 93 mm/Hg (decrease of 4 torr, −4.1%, *p* < 0.01). Average MAP decreased the most in the 1st month of RPM and continued to slowly decline over the course of the 6-month observation period. See *Table 1* and *Figure 1*.

**Table 1.** Mean Arterial Pressure Changes (mm/Hg) for Total Patient Cohort After 6 Months of Remote Physiological Monitoring

	**BASELINE**	**END**	**CHANGE**	***P***
MAP	97	93	−4%	<0.01

MAP, mean arterial pressure.

### MAP CHANGES BY INITIAL QUARTILE

**Fig. 2.**
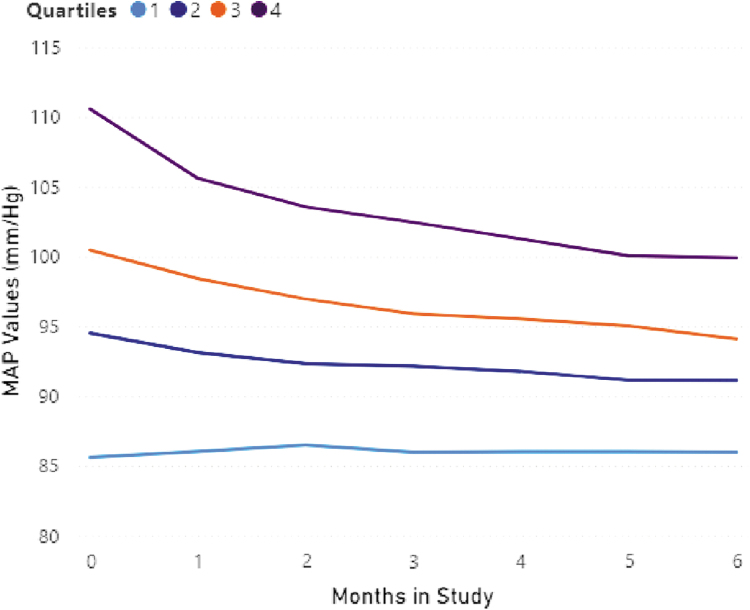
MAP (mm/Hg) changes by quartile after 6 months of RPM.

Each quartile consisted of 275 patients and was defined by average initial MAP values. Q-1, Q-2, Q-3, and Q-4 began with MAPs of 86 (normotensive), 95 (normotensive), 101 (mildly hypertensive), and 111 (hypertensive), respectively. After 6 months on the Verustat RPM program, Q-1 patients saw no change with average MAP remaining 86 (*p* = 0.42); Q-2 patients dropped average MAP 4% to 91 (*p* < 0.001); Q-3 patients dropped 6% to 94 (*p* < 0.001), and Q-4 patients dropped average MAP 10% to 100 (*p* = < 0.001). Of note, Q-3 and Q-4 patients, which comprise 50% of the cohort, improved from initially hypertensive to normotensive after 6 months of RPM. See *Table 2* and *Figure 2*.

**Table 2.** Mean Arterial Pressure (mm/Hg) Changes by Quartile After 6 Months of Remote Physiological Monitoring

QUARTILE	BASELINE	END	CHANGE	***P***
Q1	86	86	0%	0.42
Q2	95	91	−4%	0.001
Q3	100	94	−6%	0.001
Q4	111	100	−10%	0.001

### MAP CHANGES BY NUMBER OF DAYS MEASURED PER MONTH

We divided the 1,102 patients into six groups based on the number of days per month MAP measurements were reported. We then divided the patients in each of these groups into quartiles based on initial MAP.

**Fig. 3.**
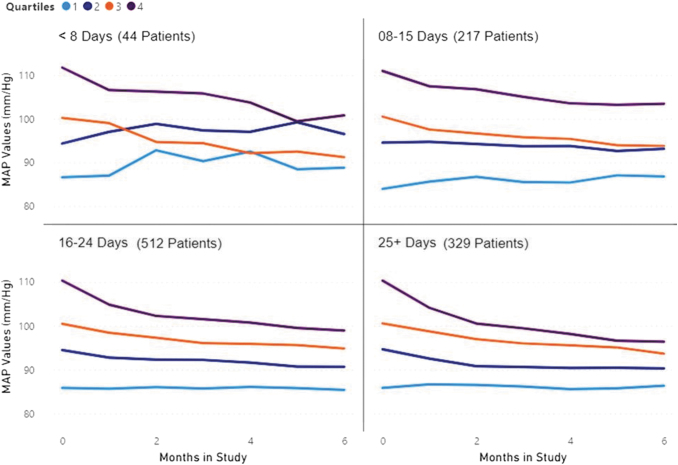
MAP (mm/Hg) by monitored days per month per quartile after 6 months of RPM.

During the first 6 months of observation, 44 patients (4%) averaged 0–7 days per month of measurements, 217 patients (20%) averaged 8–15 days per month of measurements, 512 patients (46%) averaged 16–24 days per month of measurements, and 329 patients (30%) averaged >24 days per month of measurements. This resulted in 841 patients (76%) who averaged meeting the Medicare minimum threshold for RPM reimbursement of 16 days or more days per month of measurements, whereas 261 patients (24%) did not. See *Table 3* and *Figure 3*.

**Table 3.** Mean Arterial Pressure (mm/Hg) by Monitored Days per Month per Quartile After 6 Months of Remote Physiological Monitoring

COHORTS	<8 DAYS			8–15 DAYS			16–24 DAYS			25+ DAYS		
COHORT SIZE	44 (4%)			217 (20%)			512 (46%)			329 (30%)		
QUARTILE	BASE	END	***P***	BASE	END	***P***	BASE	END	***P***	BASE	END	***P***
Q1	87	89	0.37	84	86	0.12	86	85	0.52	86	86	0.58
Q2	94	97	0.66	95	93	0.29	94	91	<0.001	95	90	<0.001
Q3	100	91	<0.001	101	94	<0.001	100	95	<0.001	101	94	<0.001
Q4	112	95	<0.001	100	96	<0.001	110	99	<0.001	110	96	<0.001
Total	101	95	0.02	100	96	<0.001	97	92	<0.001	96	91	<0.001

**Fig. 4.**
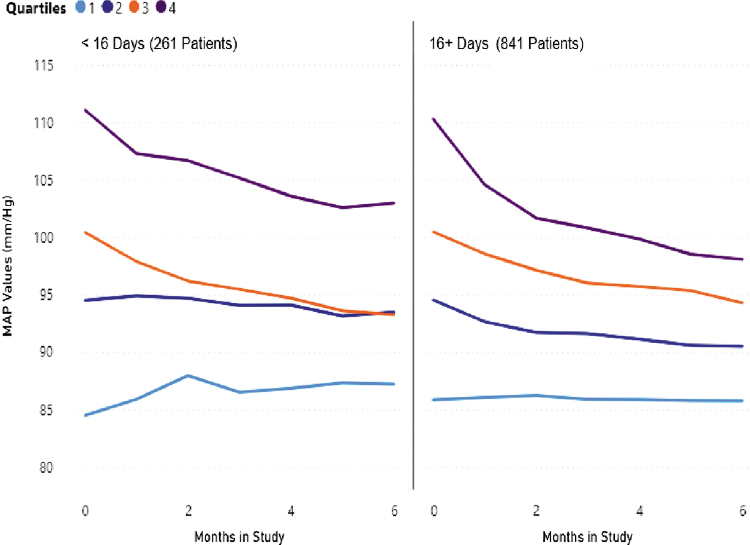
MAP changes (mm/Hg) by Medicare defined “compliant and billable” measurement days per month.

As given in *Table 4* and *Figure 4*, the more days per month the initial hypertensive patients (Q-3 and Q-4) reported BP measurements, the more their MAP declined. Patients who were initially hypertensive and measured at least 16 days per month became normotensive after 6 months of RPM.

**Table 4.** Mean Arterial Pressure Changes (mm/Hg) by Medicare Defined “Compliant and Billable” Measurement Days per Month

	<16 DAYS, NONCOMPLIANT			16+ DAYS, COMPLIANT		
	261 PATIENTS (24%)			841 PATENTS (76%)		
QUARTILE	BASE	END	***P***	BASE	END	***P***
Q1	84	88	0.08	86	86	0.88
Q2	95	93	0.41	95	91	<0.001
Q3	101	93	<0.001	101	94	<0.001
Q4	112	103	<0.001	110	98	<0.001
Total	100	96	0.00	96	91	<0.01

However, there were clinically and statistically significant MAP reductions in the hypertensive patients regardless of the number of days per month they monitored. No clearly defined minimum cut-point was found that identified a “number of measurements per month” metric below which hypertensive patients did not have a significant reduction in MAP.

## Discussion

A recently published review of the RPM literature reported that less than half of published studies showed any benefit, most showed no improvement, and a few showed worse outcomes with RPM.^5^ Although unequivocal outcomes data proving the value proposition of RPM in chronic disease management have yet to appear, we believe this report adds to the body of knowledge about RPM-associated outcomes.

This observational trial is the first to report an association of RPM with changes in average MAP as a function of the number of days of measurements per month and of initial average MAP at the start of RPM. This uncontrolled study suggests that RPM is associated with 5% average MAP reduction after 6 months of data collection and that MAP declined incrementally over this interval. Most of this reduction occurred in the 50% of patients who were hypertensive when they entered the RPM program. Of note, these hypertensive patients' average MAP became normal during the 6 months of observation.

In addition, our data suggest that there is a significant reduction in MAP among initially hypertensive patients at all levels of “days per month measurements” and that no minimum number of measurements predicts success or failure. This contrasts with the current CMS rules that require at least 16 measurements per month before a provider can deem a patient compliant with therapy and submit a bill for RPM reimbursement. We are unaware of any evidence supporting the 16 measurements per month rule, and our opinion is that CMS should consider lowering the measurements necessary for compliance to encourage wider adoption of RPM.

## Limitations

The biggest limitation to our report is its observational uncontrolled design. All patients in our study were on RPM and we report only average MAP values for the various groups, not results for individual patients. Our observation period after enrollment was 6 months and we cannot comment on the persistence of the changes we saw. Also, we do not know the mechanism responsible for the MAP drops we observed. We cannot speculate whether the changes were related to intervention by providers, improved antihypertension therapy compliance by the patients, or other unknown factors. We recognize that the small number of patients in the 0–7 and 8–15 days per month of measurements groups (44 patients, 4% of total, and 217 patients, 20% of total, respectively) weakens our conclusions regarding the effect of measurement frequency on MAP reduction.

## Conclusions

This observational report suggests that 6 months of the Verustat RPM program is associated with a clinically meaningful and statistically significant reduction in average MAP in a large cohort of patients.

This effect was largely confined to the 50% of patients whose initial MAP readings were elevated. Although more frequent days per month of BP monitoring was associated with more robust MAP reduction in these patients, no minimum number of days per month of measurements was seen below which the reduction disappeared. Large randomized controlled trials using endpoints such as mortality, hospitalizations, health care spending, and chronic disease complications are needed to prove the value of RPM in managing chronic diseases.

## Disclaimer

As submitting author, I attest that I have written consent from all authors to submit the article, and that all authors accept complete responsibility for the contents of this article. This article has not been previously published. Finally, I attest that the article is independent and original work, and all conclusions are from the authors. At the time of its writing, Dr. Frazier was employed by Verustat Inc., which funded the study. The other four authors were employed by Dobson DaVanzo and Associates LLC that has no financial interests in Verustat, Inc.

## References

[1] Telehealth and remote patient monitoring—HHS.gov. Available from: https://telehealth.hhs.gov/providers/preparing-patients-for-telehealth/telehealth-and-remote-patient-monitoring/ [Last accessed: April 22, 2022].

[2] Bayliss EA, Steiner JF, Fernald DH, et al. Descriptions of barriers to self-care by persons with comorbid chronic diseases. Ann Fam Med 2003;1(1):15–21; doi: 10.1370/afm.4 15043175 PMC1466563

[3] Coye MJ, Haselkorn A, DeMello S. Remote patient management: Technology-enabled innovation and evolving business models for chronic disease care. Health Aff (Millwood) 2009;28(1):126–135; doi: 10.1377/hlthaff.28.1.126 19124862

[4] Medicare program; revisions to payment policies under the Physician Fee Schedule and other revisions to Part B for CY 2019; Medicare Shared Savings Program requirements; Quality Payment Program; Medicaid Promoting Interoperability Program; Quality Payment Program—extreme and uncontrollable circumstance policy for the 2019 MIPS payment year; Provisions from the Medicare Shared Savings Program—accountable care organizations—pathways to success; and expanding the use of telehealth services for the treatment of opioid use disorder under the Substance Use-Disorder Prevention that Promotes Opioid Recovery and Treatment (SUPPORT) for Patients and Communities Act. Federal Register. November 23, 2018. Available from: https://www.federalregister.gov/documents/2018/11/23/2018-24170/medicare-program-revisions-to-payment-policies-under-the-physician-fee-schedule-and-other-revisions#h-81 [Last accessed: August 5, 2022].

[5] Walker RC, Tong A, Howard K, et al. Patient expectations and experiences of remote monitoring for chronic diseases: Systematic review and thematic synthesis of qualitative studies. Int J Med Inform 2019;124:78–85; doi: 10.1016/j.ijmedinf.2019.01.013 30784430

